# *Kdf1* Regulates Molar Cusp Morphogenesis via the PI3K/AKT/mTOR Signalling Axis

**DOI:** 10.1111/cpr.70108

**Published:** 2025-07-30

**Authors:** Jiayu Wang, Miao Yu, Hangbo Liu, Kai Sun, Chenxin Geng, Haochen Liu, Hailan Feng, Yang Liu, Hu Zhao, Dong Han

**Affiliations:** ^1^ Department of Prosthodontics Peking University School and Hospital of Stomatology, National Center for Stomatology, National Clinical Research Center for Oral Diseases, National Engineering Research Center of Oral Biomaterials and Digital Medical Devices Beijing China; ^2^ Chinese Institute for Brain Research Beijing China

**Keywords:** cell proliferation, inner enamel epithelium, *Kdf1*, PI3K/AKT signalling pathway, tooth cusp morphogenesis

## Abstract

*Keratinocyte differentiation factor 1* (*Kdf1*) reportedly plays a significant role in enamel formation. In terms of tooth morphogenesis, human *KDF1* variants are associated with crown morphological abnormalities, suggesting that *Kdf1* may also be essential for tooth morphogenesis. However, the involvement of *Kdf1* in tooth morphogenesis and its underlying mechanisms remains unclear. In this study, we observed that mice lacking epithelial *Kdf1* (*K14‐Cre;Kdf1* ^*fl/fl*^) displayed rounded and blunt molar cusps, resembling the morphological anomalies observed in patients with *Kdf1* variants. 5‐Ethynyl‐2′‐deoxyuridine assays revealed increased proliferative activity of the inner enamel epithelial (IEE) cells in the cusp region of *K14‐Cre;Kdf1* ^*fl/fl*^ mice during the bell stage. RNA sequencing and western blot analysis confirmed the overactivation of PI3K/AKT/mTOR signalling in the molar IEE cells of *K14‐Cre;Kdf1* ^*fl/fl*^ mice. Furthermore, in utero microcapillary injection of the PI3K/AKT/mTOR pathway inhibitor LY294002 partially rescued the molar cusp defects in *K14‐Cre;Kdf1* ^*fl/fl*^ mice. Collectively, our findings provide in vivo evidence supporting the regulatory role of *Kdf1* in molar cusp morphogenesis, highlighting its function in modulating dental epithelial cell proliferation via the PI3K/AKT/mTOR signalling pathway.

## Graphical abstract

Epithelial *Kdf1* knockout disrupts molar cusp morphogenesis by promoting inner enamel epithelium proliferation and invagination via PI3K/AKT/mTOR signalling.

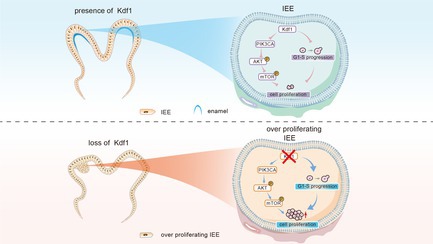

## 1 Introduction

The cusp is a prominent structure of the molar crown that plays a critical role in determining the overall crown shape and facilitating food grinding during mastication [1]. Molar cusps with optimally shaped exhibit enhanced occlusal efficiency [2, 3]. The morphology of these cusps is regulated by a highly coordinated process involving the sequential activation of enamel knots, signalling pathways within the epithelium and interactions between epithelial and mesenchymal tissues [4]. Key epithelial signals, including bone morphogenetic proteins, fibroblast growth factors, sonic hedgehog and wingless‐type MMTV integration site family (Wnt) signalling pathways, are essential for the induction of molar cusp formation [5, 6, 7, 8, 9].

During the progression of tooth development, the dental epithelium undergoes proliferation and expansion around the dental mesenchyme, shaping the tooth through the bud, cap, and bell stages [10]. In the late cap stage, the inner enamel epithelium (IEE), formed from the inner cuboidal cells of the enamel organ, transitions into a columnar shape [11, 12]. The IEE is crucial in defining the shape of the tooth crown and ultimately differentiates into ameloblasts, which are responsible for enamel formation. Evidence indicates that transgenic mice with tooth cusp developmental abnormalities exhibit abnormal proliferation of the IEE [13, 14, 15], pointing to a close relationship between cusp morphology and the regulation of IEE cell proliferation. These findings support the notion that the proliferation of IEE cells plays a significant role in tooth cusp morphogenesis.

The *keratinocyte differentiation factor 1* (*Kdf1*) gene was first identified and characterised in mice through an unbiased *N*‐ethyl‐*N*‐nitrosourea (ENU)‐induced mutagenesis screen [16]. Lee et al. found that *Kdf1* mutant mice (*shd* mice) exhibited a thickened and tightly bound epidermis with impaired barrier function. They demonstrated that *Kdf1* inhibits epidermal progenitor cell proliferation by regulating the transcription factor *p63* [16]. Recently, accumulating evidence has further elucidated the role of *Kdf1* in tooth development. The human *KDF1* gene has been implicated as a pathogenic factor in hypohidrotic ectodermal dysplasia [17] and non‐syndromic tooth agenesis [18, 19]. Notably, we recently reported abnormal tooth crown morphology in a non‐syndromic tooth agenesis pedigree associated with a *KDF1* variation (c.920G>C, p.R307P), presenting with rounded and blunt cusps, as well as flattened grooves in the premolars and molars [18].

In murine models, Zeng et al. reported that Kdf1 protein is specifically expressed in the dental epithelium, but not in the mesenchyme, during early embryonic development [19]. Knock‐in mice harbouring a missense variant of *Kdf1* (c.908G>C, p.R303P) displayed enamel structural defects, indicating that *Kdf1* plays a crucial role in tooth enamel formation [20]. Our previous in vitro studies further demonstrated that *Kdf1* promotes ameloblast differentiation through the IKK/IκB/NF‐κB signalling pathway, highlighting its essential role in enamel mineralisation [21]. However, it remains unclear whether *Kdf1* is directly involved in tooth crown morphogenesis. Further studies are needed to fully understand the functional role of *Kdf1* in shaping tooth development.

The PI3K/AKT/mTOR signalling pathway is a highly conserved signal transduction mechanism across eukaryotic cells, playing a critical role in cellular growth, proliferation and survival [22, 23, 24]. This pathway also has significant involvements in tooth development [25, 26]. Studies on the molars of miniature pigs have demonstrated that the PI3K/AKT pathway is initially activated in the IEE and subsequently triggers activation in the dental papilla, promoting the differentiation of odontoblasts [26]. Additionally, mTOR, a key downstream effector of the PI3K/AKT signalling cascade, exerts multiple regulatory effects on the development of both the dental epithelium and mesenchyme [25, 27, 28]. Conditional suppression of mTOR signalling in the dental epithelium of mice leads to hypoplastic molars with atypical cusp morphology [27]. Conversely, the deletion of mTOR in cranial neural crest cells—which primarily give rise to the mesenchymal compartment of the tooth germ—results in the arrest of tooth development at the bud stage [28]. Interestingly, mTOR signalling ablation in odontoblasts enhances mineralisation in vivo, leading to increased dentin thickness [25]. Although it has been reported that the PI3K/AKT signalling pathway is activated in cervical cancer cells with *KDF1* variants [29, 30], it remains unclear whether this pathway, and its downstream effectors, are altered in the teeth of *Kdf1* deficient mice and how such alterations might influence tooth development.

In this study, we generated epithelial *Kdf1* specific knockout mice, *K14‐Cre;Kdf1* ^*fl/fl*^ and investigated the morphological defects of the molar crown and alterations in the proliferation of IEE cells at molar cusps during early tooth development to explore the molecular mechanism of epithelial *Kdf1* in modulating IEE cell proliferation during molar cusp morphogenesis.

## 2 Materials and Methods

### 2.1 Mouse Strains

We developed *K14‐Cre;Kdf1* ^*fl/fl*^ mice by crossing *K14‐Cre* [31] and *Kdf1* ^*fl/fl*^ (generated by Cyagen, Guangzhou, China) mouse lines with a C57BL/6J background. The mice were housed under specific pathogen‐free conditions. All procedures involving mice were approved by the Ethics Committee of the Peking University Health Science Center (LA2022177).

### 2.2 Tooth Germ Preparation and RNAscope In Situ Hybridisation

On the basis of the appearance of the vaginal plug, timed‐pregnant C57BL/6J mice (Department of Laboratory Animal Science, Peking University Health Science Center, Beijing, China) were sacrificed at the stages of embryonic (E) day 11.5 (E11.5), E12.5, E13.5, E14.5, E15.5, E16.5, E17.5, E18.5 and PN0.5, respectively. The heads of mice corresponding to each embryonic stage were dissected and fixed in 4% paraformaldehyde (PFA) overnight, demineralised with EDTA calcifying solution, dehydrated in graded ethanol series, paraffin embedded, and sectioned serially (5 μm). RNAscope Probe‐Mm‐Kdf1 (ACD‐1029511; Advanced Cell Diagnostics, San Francisco, CA, USA) was used to detect the *Kdf1* mRNA with RNAscope 2.5 HD Reagent Kit‐Red (ACD‐322350; Advanced Cell Diagnostics) following the manufacturer's procedure [32].

### 2.3 Whole Mount Skeleton Staining With Alizarin Red and Alcian Blue

To visualise the skeleton, mice were sacrificed at PN0.5 and stained with alcian blue and alizarin red (Solarbio, Beijing, China), as previously described [33].

### 2.4 Kidney Capsule Transplantation

The mandibular first molar germs of *K14‐Cre;Kdf1* ^*fl/fl*^ and *Kdf1* ^*fl/fl*^ mice at PN0.5 were transplanted beneath the kidney capsule of C57BL/6J male mice [34]. After 2 weeks of growth in the kidney capsule, the mouse kidneys were harvested to examine the molar cusp morphology.

### 2.5 Micro‐Computed Tomography

Histomorphometry was conducted on the mandibular first molar after renal subcapsular development using Siemens Inveon micro‐Computed Tomography (CT) (Siemens Medical Solutions USA Inc., Malvern, PA, USA) at 60 kV and 33 pixels. The 3D images were reconstructed using Avatar software (PINGSENG Healthcare, Kunshan, China).

### 2.6 Histologic Analysis

Heads of embryos (E13.5, E15.5, E16.5, E17.5 and E18.5) and newborn mice were fixed in 4% PFA solution overnight at 4°C. After dehydration through graded alcohols, tissues were embedded in paraffin wax and sectioned at 5 μm. Standard procedures were adopted for H&E staining [35].

### 2.7 5‐Ethynyl‐2′‐Deoxyuridine Incorporation and Staining

5‐Ethynyl‐2′‐deoxyuridine (EdU) (25 mg/g; RiboBio) was intraperitoneally injected into pregnant mice (13.5, 15.5, 16.5, 17.5 and 18.5 days) or newborn mice 3 h prior to euthanisation, and the incorporated EdU was detectable by the Click‐iT Apollo 567 Stain Kit (RiboBio). For each time point, littermate‐matched *Kdf1* ^*fl/fl*^ and *K14‐Cre;Kdf1* ^*fl/fl*^ mice were collected as biological replicates (*n* = 3 pairs per time point).

### 2.8 Immunofluorescence Assays

Paraffin sections at 5 μm from embryos (E13.5, E15.5, E16.5, E17.5 and E18.5) and newborn mice were processed and used for the immunofluorescence staining.

### 2.9 Laser Capture Microdissection

We used laser capture microdissection (LCM) to obtain the IEE of the mandibular first molars from the head sections of E18.5 mice with high spatial resolution. After washing with ice‐cold RNAse‐free PBS, the embryo head was immediately frozen at −80°C in tissue freezing medium (Leica Microsystems, Wetzlar, Germany) and sliced at 20 μm thickness (Leica Microsystems). The sections were fixed in ice‐cold acetone for 1 min. The IEE area of the mandibular first molar was captured using a LCM system (Leica Microsystems, Germany), and tissues were collected for RNA sequencing (RNA‐seq) and western blotting.

### 2.10 RNA‐Seq and Data Analysis

RNA samples from the IEE of the mandibular first molars of *Kdf1* ^*fl/fl*^ and *K14‐Cre;Kdf1* ^*fl/fl*^ mice at the E18.5 stage (*n* = 3 per group) were used for RNA‐seq.

### 2.11 Western Blot

Total protein was extracted from the IEE using RIPA buffer (Beyotime, China). Protein lysates were resolved by SDS–PAGE and transferred onto a nitrocellulose membrane. Blots were probed with primary antibody against Kdf1, AKT, p‐AKT (Ser473), mTOR, p‐mTOR (Ser2448), PIK3CA, GSK3β, p‐GSK3β (Ser9), FOXO3a, p‐FOXO3a (S253) or β‐actin.

### 2.12 In Utero Microinjection With Microcapillary

For the rescue experiment, we used microcapillary tubes, with reference to previous methods [36, 37] to inject the PI3K/AKT/mTOR pathway inhibitor (LY294002, Selleck, Houston, USA) into the mandibular molar alveolar fossa region of E17.5 mouse embryos under a microscope in utero. Pregnant mice were anaesthetised using isoflurane (5% for induction and 2% for maintenance). Surgical hair clipping around the umbilicus was performed, followed by an alternative alcohol–iodine treatment to prepare an aseptic surgical zone. A midsagittal incision was gently made using forceps and scissors, and one uterine horn was exposed using the forceps. Subsequently, all horns were carefully pulled out from one side up to the ovary, revealing all embryos. In mice, the mandibular first molar is located approximately below the eyes [38]. To inject the inhibitor LY294002 into the alveolar fossa while avoiding damage to the molar germs, we aimed the microcapillary towards the mouse eye, inserted the tips through the uterine wall, placed the tips on the horizontal surface of the mandible, and then inserted the tips into the cortical bone under a microscope. Subsequently, 3 μL of LY294002 at a concentration of 3 mg/mL was injected into the left mandibular molar alveolar fossa region of all embryos. An equal volume of DMSO was injected into the alveolar fossa region of the right mandibular molar of all embryos as a control. Finally, layered sutures were placed using resorbable sutures in the operative area, followed by post‐surgical and follow‐up care. Two days after surgery, pregnant mice were sacrificed, and E19.5 mouse embryos were harvested for H&E and immunofluorescence staining of cyclin D1. IEE tissue from E19.5 mouse embryos was collected by LCM, as described prior, and used for western blot analysis.

### 2.13 Statistical Analysis

All experiments were repeated at least three times, and representative images were obtained for the publication. All data were presented as mean ± SD. GraphPad Prism10 was used for statistical analysis. Student's *t*‐test was used to assess statistical significance. *p* values less than 0.05 were considered statistically significant in all comparisons.

## 3 Results

### 3.1 Kdf1 Was Specifically Expressed in the Epithelium During Tooth Development

**FIGURE 1:**
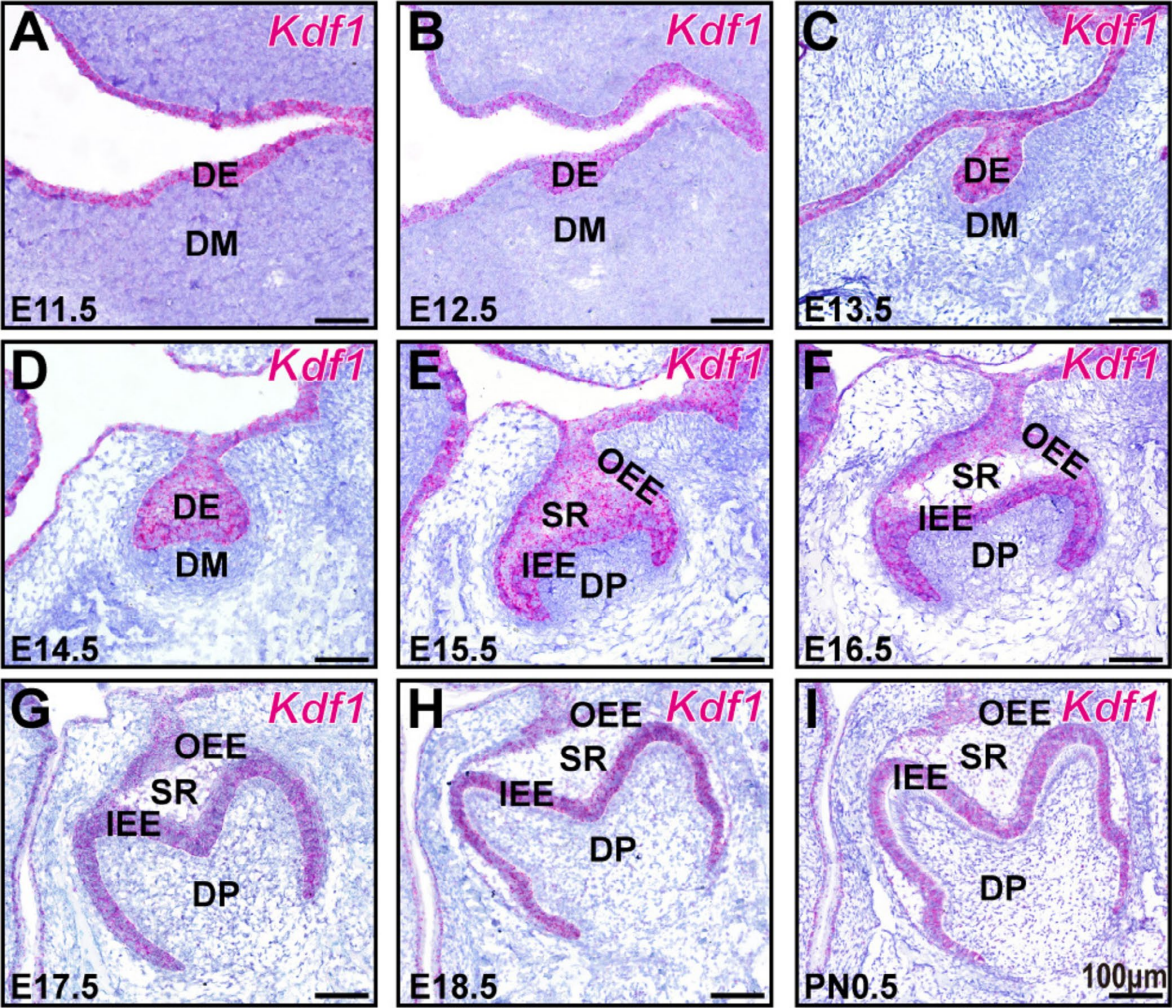
Expression pattern of *Kdf1* transcripts during early molar development. (A–I) RNAscope in situ hybridization of *Kdf1* transcript signals from wild‐type mice with positive staining in red of the coronal sections of MM1 from E11.5 to PN0.5. Scale bars: 100 μm. DE, dental epithelium; DM, dental mesenchyme; DP, dental pulp; IEE, inner enamel epithelium; OEE, outer enamel epithelium; SR, stellate reticulum. *n* = 3 per group.

To investigate the role of *Kdf1* in regulating molar morphogenesis, we first determined the *Kdf1* expression patterns during early tooth development at the transcriptional level using RNAscope hybridisation. At the dental lamina stage (E11.5), *Kdf1* was expressed in the dental lamina and oral epithelium (Figure 1A). From the bud stage to the cap stage (E12.5–E15.5), *Kdf1* was present in the enamel organ epithelium and oral epithelium (Figure 1B–E). At the bell stage (E16.5–PN0.5), *Kdf1* was still present in the enamel organ epithelium and gradually concentrated towards the IEE (Figure 1F–I).

### 3.2 Generation of Epithelial Kdf1 Knockout Mice and Associated Epidermal Morphological Defects

**FIGURE 2:**
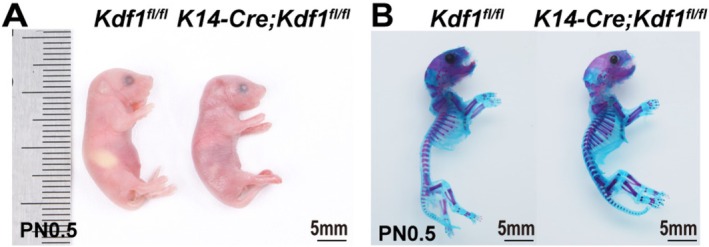
Morphological defects in epidermis of *K14‐Cre;Kdf1* ^*fl/fl*^ mice. (A) Gross morphological comparison of *Kdf1* ^*fl/fl*^ and *K14‐Cre;Kdf1* ^*fl/fl*^ mice at PN0.5. Scale bars: 5 mm. (B) Gross skeletal morphological comparison of *Kdf1* ^*fl/fl*^ and *K14‐Cre;Kdf1* ^*fl/fl*^ mice at PN0.5. Scale bars: 5 mm. *n* = 3 per group.

Given this unique expression pattern of the *Kdf1* gene, we generated epithelial *Kdf1* knockout mice, *K14‐Cre;Kdf1* ^*fl/fl*^ (Figure S1A,B). Western blotting of the mandibular first molar (MM1) germs from *Kdf1* ^*fl/fl*^ and *K14‐Cre;Kdf1* ^*fl/fl*^ mice at PN0.5 confirmed the knockout efficiency of Kdf1 in the dental epithelium of *K14‐Cre;Kdf1* ^*fl/fl*^ mice (Figure S1C). *K14‐Cre;Kdf1* ^*fl/fl*^ mice died shortly after birth. At this stage, these mice exhibited smooth, taut skin covering the entire body and were unable to open their mouths (Figure 2A). Skeleton staining revealed no significant differences in the gross skeletal morphology between *K14‐Cre;Kdf1* ^*fl/fl*^ and *Kdf1* ^*fl/fl*^ mice at PN0.5 (Figure 2B). These findings suggested that the epithelial‐specific ablation of *Kdf1* primarily disrupts epidermal development without affecting overall skeletal formation.

### 3.3 Ablation of Epithelial Kdf1 Altered the Morphology of Molar Cusps

**FIGURE 3:**
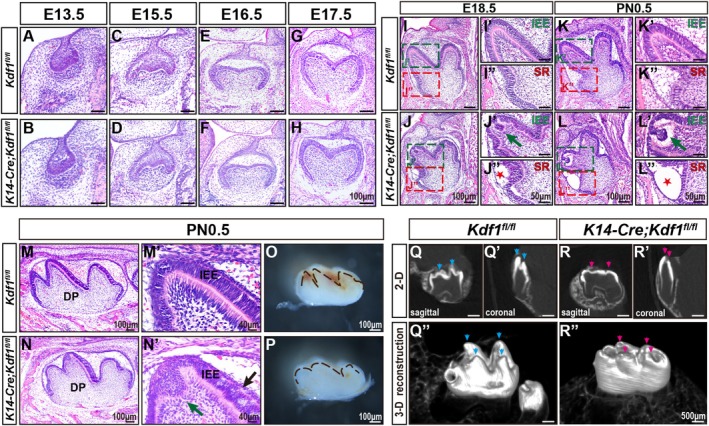
Altered cusp morphology in mandibular first molar of *K14‐Cre;Kdf1* ^*fl/fl*^ mice. (A–L) Representative coronal images of H&E stained MM1 from *Kdf1* ^*fl/fl*^ and *K14‐Cre;Kdf1* ^*fl/fl*^ mice at E13.5, E15.5, E16.5, E17.5, E18.5 and PN0.5. Scale bars: 100 μm. (I′–L′) Higher magnification of the green dashed boxes in I–L at E18.5 and PN0.5. Green arrows indicate the aberrant folding of the IEE from *K14‐Cre;Kdf1* ^*fl/fl*^ mice in J′ and L′. Scale bars: 50 μm. (I″–L″) Higher magnification of the red dashed boxes in I–L at E18.5 and PN0.5. Red asterisks indicate the excessively loose morphology of the stellate reticulum of *K14‐Cre;Kdf1* ^*fl/fl*^ mice in J″ and L″. Scale bars: 50 μm. (M, N) Sagittal images of HE‐stained MM1 from *Kdf1* ^*fl/fl*^ and *K14‐Cre;Kdf1* ^*fl/fl*^ mice at PN0.5. Scale bars: 100 μm. (M′, N′) Higher magnification images of cusp region in M and N. Green arrow indicates aberrant folding of the IEE from *K14‐Cre;Kdf1* ^*fl/fl*^ mice in N′. Black arrow shows the morphology of the IEE cells from *K14‐Cre;Kdf1* ^*fl/fl*^ mice in N′. Scale bars: 40 μm. (O, P) The stereomicroscopic views of MM1 from *Kdf1* ^*fl/fl*^ and *K14‐Cre;Kdf1* ^*fl/fl*^ mice at PN0.5 on a sagittal plane. The brown dashed lines outline the cusp morphology. Scale bars: 100 μm. (Q, R, Q′, R′, Q″, R″) Representative micro–computed tomography (μCT) images of MM1 of *Kdf1* ^*fl/fl*^ and *K14‐Cre;Kdf1* ^*fl/fl*^ mice cultured under the kidney capsule from sagittal (Q, R), coronal (Q′, R′) and 3D reconstruction (Q″, R″) views. Blue arrowheads show the cusps of *Kdf1* ^*fl/fl*^ mice, red arrowheads show the cusps of *K14‐Cre;Kdf1* ^*fl/fl*^ mice. Scale bars: 500 μm. DP, dental pulp; IEE, inner enamel epithelium; SR, stellate reticulum. *n* = 3 per group.

To determine whether epithelial *Kdf1* ablation affects the morphogenesis of molars, we analysed the morphology of MM1 germs. From E13.5 to E17.5, H&E staining on coronal sections of MM1 germs of *K14‐Cre;Kdf1* ^*fl/fl*^ mice showed similar crown morphology compared with those of *Kdf1* ^*fl/fl*^ mice (Figure 3A–H). At E18.5 and PN0.5, on coronal sections, H&E staining results showed obvious abnormalities in the developing cusp region compared with that of *Kdf1* ^*fl/fl*^ mice (Figure 3I–L). Specifically, the cusps of MM1 in *K14‐Cre;Kdf1* ^*fl/fl*^ mice showed shallow and blunt shapes with aberrant folding of IEE towards the dental pulp mesenchyme (Figure 3J′,L′), whereas MM1 in *Kdf1* ^*fl/fl*^ mice showed normal morphology, presenting with sharp cusps and deep grooves (Figure 3I′,K′). In addition, at E18.5 and PN0.5, the morphology of the stellate reticulum of MM1 of *K14‐Cre;Kdf1* ^*fl/fl*^ mice became excessively loose, causing a vacant part (Figure 3J″,L″), whereas the stellate reticulum of *Kdf1* ^*fl/fl*^ mice showed regular morphology with star shape (Figure 3I″,K″). Consistently, in the sagittal view, the cusp morphologies of MM1 in *K14‐Cre;Kdf1* ^*fl/fl*^ and *Kdf1* ^*fl/fl*^ mice at PN0.5 exhibited similar results (Figure 3M,N,M′,N′). Moreover, the shallow and blunt shapes of MM1 cusp in *K14‐Cre;Kdf1* ^*fl/fl*^ mice were also confirmed by gross observations of the sagittal view under a stereomicroscope, comparing to the regular cusp morphology in *Kdf1* ^*fl/fl*^ mice (Figure 3O,P). These data indicate that epithelial‐specific ablation of *Kdf1* leads to molar cusp malformation, starting around the late bell stage (E18.5).

Because *K14‐Cre;Kdf1* ^*fl/fl*^ mice died shortly after birth due to the dysplasia of all epithelium‐derived organs, we conducted kidney capsule transplantation of MM1 germs from PN0.5 mice to explore the possible morphological impact of mature crown caused by epithelial *Kdf1* knockout. After 2 weeks of culture in vivo, micro‐CT scanning and 3D reconstruction showed that the mature MM1 crown of *K14‐Cre;Kdf1* ^*fl/fl*^ mice exhibited flat grooves, and the cusps were rounded and blunt (Figure 3R,R′,R″), compared with the mature MM1 crown of *Kdf1* ^*fl/fl*^ mice (Figure 3O,O′,O″). These results confirmed that ablation of *Kdf1* in the dental epithelium leads to the eventual malformation of molar cusps. Taken together, our data suggest that *Kdf1* plays a crucial role in regulating molar cusp development.

### 3.4 Ablation of Epithelial Kdf1 Resulted in the Exorbitant Proliferation of IEE Cells

**FIGURE 4:**
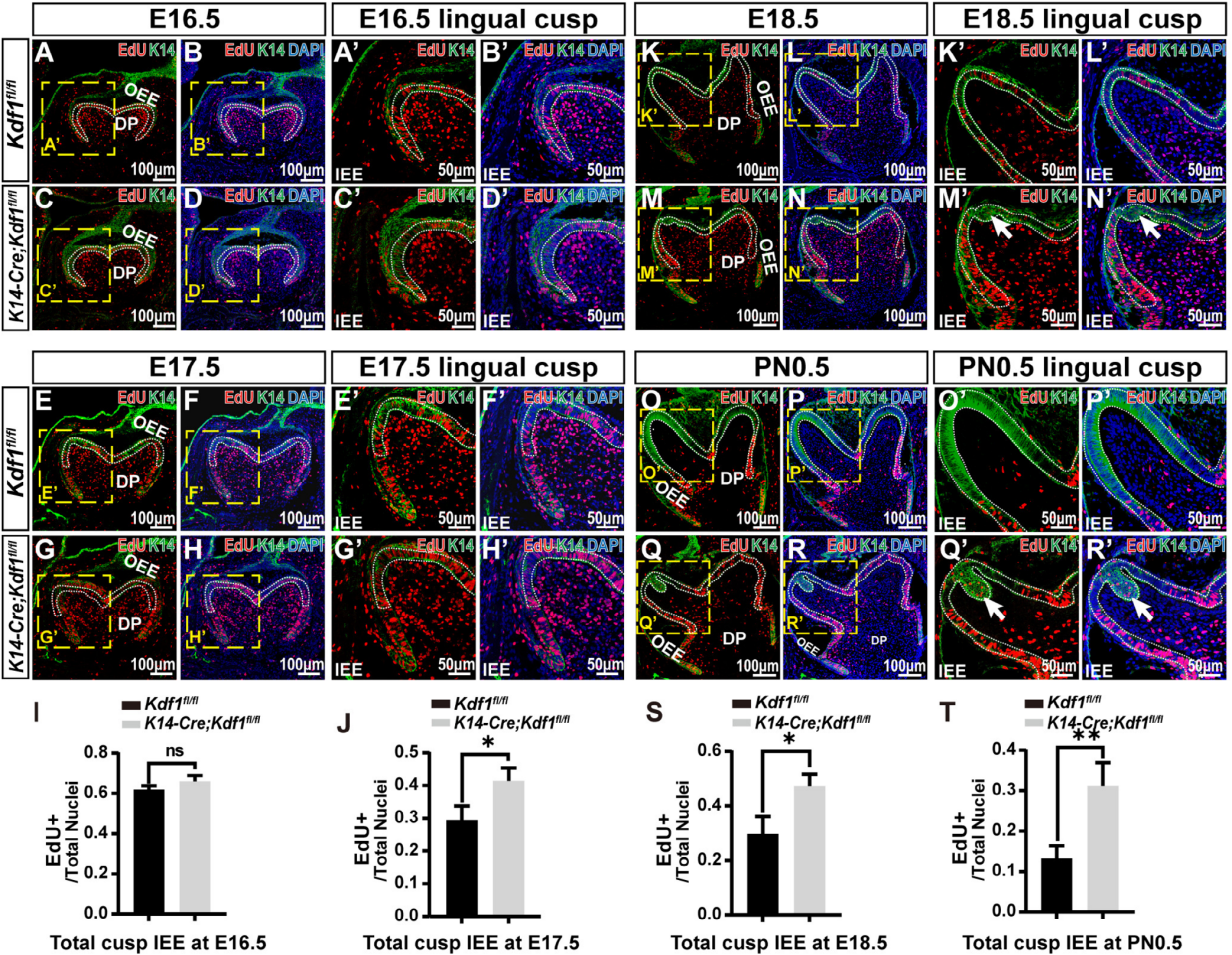
Exorbitant proliferation activity of cusp IEE cells in *K14‐Cre;Kdf1* ^*fl/fl*^ mice from E17.5. (A–H) Combinations of three fluorescence signals (anti‐K14 in green, EdU in red, and DAPI in blue) show EdU‐positive cells of MM1 from *Kdf1* ^*fl/fl*^ and *K14‐Cre;Kdf1* ^*fl/fl*^ mice at E16.5 and E17.5. The white dashed lines circle the IEE at the cusp region. Scale bars: 100 μm. (A′–H′) Higher magnification of the yellow dashed boxes in A–H. The white dashed lines circle the cusp IEE. Scale bars: 50 μm. (I, J) Ratios of EdU^+^/DAPI^+^ cells exhibit the cell proliferation rate of entire cusp IEE at E16.5 and E17.5. (K–R) Combinations of three fluorescence signals (anti‐K14 in green, EdU in red, and DAPI in blue) show EdU‐positive cells of MM1 from *Kdf1* ^*fl/fl*^ and *K14‐Cre;Kdf1* ^*fl/fl*^ mice at E18.5 and PN0.5. The white dashed lines circle the IEE at the cusp region. Scale bars: 100 μm. (K′–R′) Higher magnification of the yellow dashed boxes in K–R. The white dashed lines circle the cusp IEE. Arrows indicate the EdU‐positive cells of the IEE at cusp tip from *K14‐Cre;Kdf1* ^*fl/fl*^ mice in M′, N′ and Q′, R′. Scale bars: 50 μm. (S–T) Ratios of EdU^+^/DAPI^+^ cells exhibit the cell proliferation rate of entire cusp IEE at E18.5 and PN0.5. DP, dental pulp; IEE, inner enamel epithelium; OEE, outer enamel epithelium. Values are presented as mean ± SD. ns, not significant, *p < 0.05, **p < 0.01. *n* = 3 per group.

To explore the cellular mechanisms responsible for the cusp developmental defects of MM1 in *K14‐Cre;Kdf1* ^*fl/fl*^ mice, we performed EdU staining and immunostaining to examine the proliferative activity of dental epithelial cells from E13.5 to PN0.5 (Figure S2A–D,A′–D′ and Figure 4A–H,A′–H′,K–R,K′–R′). Immunofluorescence staining of Keratin 14 was used to mark epithelial cells and distinguish the dental epithelium from the dental mesenchyme. At E13.5 and E15.5, no statistical difference was observed in the proliferation of total dental epithelial cells between *K14‐Cre;Kdf1* ^*fl/fl*^ and littermate control *Kdf1* ^*fl/fl*^ mice (Figure S2E). At E16.5, no statistical differences were observed in the total cusp IEE between the two groups (Figure 4I). At E17.5, E18.5 and PN0.5, the proliferative activity of IEE cells in the cusp region was markedly increased in *K14‐Cre;Kdf1* ^*fl/fl*^ mice compared with that in *Kdf1* ^*fl/fl*^ mice (Figure 4J,S,T). Notably, at PN0.5, no EdU^+^ cells were detected in the apex of cusp in *Kdf1* ^*fl/fl*^ mice (Figure 4O′,P′), whereas a number of EdU^+^ cells were present in the aberrant folding area of IEE in the apex of cusp in *K14‐Cre;Kdf1* ^*fl/fl*^ mice (Figure 4Q′,R′). These data demonstrate that ablation of epithelial *Kdf1* results in the exorbitant proliferation of IEE cells in the molar cusps from E17.5.

In addition, the expression of amelogenin, a marker of ameloblast differentiation, was significantly decreased in IEE cells of MM1 cusps in *K14‐Cre;Kdf1* ^*fl/fl*^ mice at PN0.5 compared to that in *Kdf1* ^*fl/fl*^ mice (Figure S3A–E), confirming that the absence of epithelial *Kdf1* suppressed the differentiation of IEE cells in the molar cusps.

### 3.5 Ablation of Epithelial Kdf1 Overactivated PI3K/AKT/mTOR Signalling in IEE Cells

**FIGURE 5:**
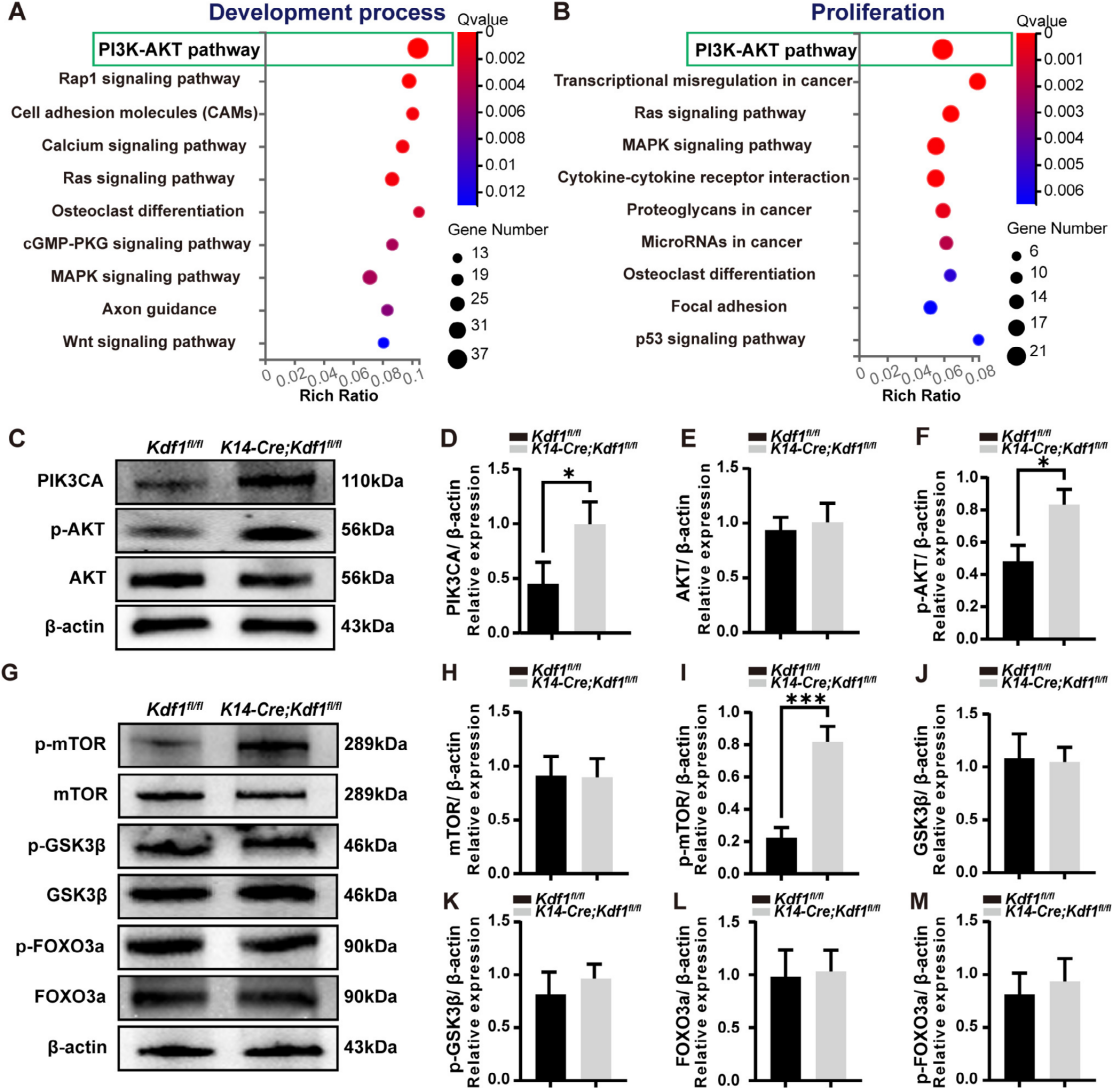
Epithelial *Kdf1* deletion activates the PI3K/AKT/mTOR signalling pathway. (A) The top 10 significantly enriched pathways in the IEE of *K14‐Cre;Kdf1* ^*fl/fl*^ molars (compared to *Kdf1* ^*fl/fl*^ molars) at E18.5 from the KEGG enrichment analysis on the filtered differential genes in development process gene sets. The top‐ranked pathway is highlighted with a green box. (B) The top 10 significantly enriched pathways in the IEE of *K14‐Cre;Kdf1* ^*fl/fl*^ molars (compared to *Kdf1* ^*fl/fl*^ molars) at E18.5 from the KEGG enrichment analysis on the filtered differential genes in proliferation process gene sets. The top‐ranked pathway highlighted with a green box. (C–F) Western blot analysis and the semi‐quantification results show the expression levels of PIK3CA, phosphor‐AKT (p‐AKT) and AKT in the IEE of *Kdf1I* ^*fl/fl*^ and *K14‐Cre;Kdf1* ^*fl/fl*^ molars at E18.5. (G–M) Western blot analysis and the semi‐quantification results show the expression levels of phosphor‐mTOR (p‐mTOR), mTOR, phosphor‐GSK3β (p‐GSK3β), GSK3β, phosphor‐FOXO3a (p‐FOXO3a) and FOXO3a in the IEE of *Kdf1* ^*fl/fl*^ and *K14‐Cre;Kdf1* ^*fl/fl*^ molars at E18.5. Values are presented as mean ± SD. *p < 0.05, ***p < 0.001. *n* = 3 per group.

To investigate the molecular mechanism underlying cusp malformation in *K14‐Cre;Kdf1* ^*fl/fl*^ mice, we performed RNA‐seq analysis of the dental epithelium dissected from the MM1 of *K14‐Cre;Kdf1* ^*fl/fl*^ mice at E18.5. In total, 993 differentially expressed genes were filtered, and gene sets related to development and proliferation were used to screen the downstream signalling pathways of *Kdf1*. Using KEGG analysis, we found that the PI3K/AKT pathway was highly enriched in both the development and proliferation gene sets (Figure 5A,B). Western blot of the core molecules in PI3K/AKT signalling and densitometry analysis results showed that the expression levels of PIK3CA and p‐AKT were both significantly increased in MM1 IEE tissues of *K14‐Cre;Kdf1* ^*fl/fl*^ mice compared with that in *Kdf1* ^*fl/fl*^ mice (Figure 5C–F). These results revealed that the PI3K/AKT pathway was overactivated in the IEE of MM1 in *K14‐Cre;Kdf1* ^*fl/fl*^ mice.

Furthermore, to investigate the influence of overactivated PI3K/AKT signalling on its downstream effector molecules, we detected the phosphorylation levels of GSK3β, FOXO3a, and mTOR, which are three important phosphorylation targets of AKT in regulating the cell proliferation process [39]. Western blot and the densitometry analysis results showed that the phosphorylation levels of mTOR were significantly increased in IEE of MM1 in *K14‐Cre;Kdf1* ^*fl/fl*^ mice (Figure 5G,H); however, there were no significant differences in p‐GSK3β and p‐FOXO3a between *Kdf1* ^*fl/fl*^ and *K14‐Cre;Kdf1* ^*fl/fl*^ mice (Figure 5G,I–M). This result suggests that the ablation of epithelial *Kdf1* resulted in the overactivation of PI3K/AKT/mTOR signalling in IEE cells of *K14‐Cre;Kdf1* ^*fl/fl*^ mice.

### 3.6 Inhibition of Overactivated PI3K/AKT/mTOR Signalling Partially Rescued the Morphological Defects of Molar Cusps in *K14‐Cre;Kdf1* ^*fl/fl*^ Mice

**FIGURE 6:**
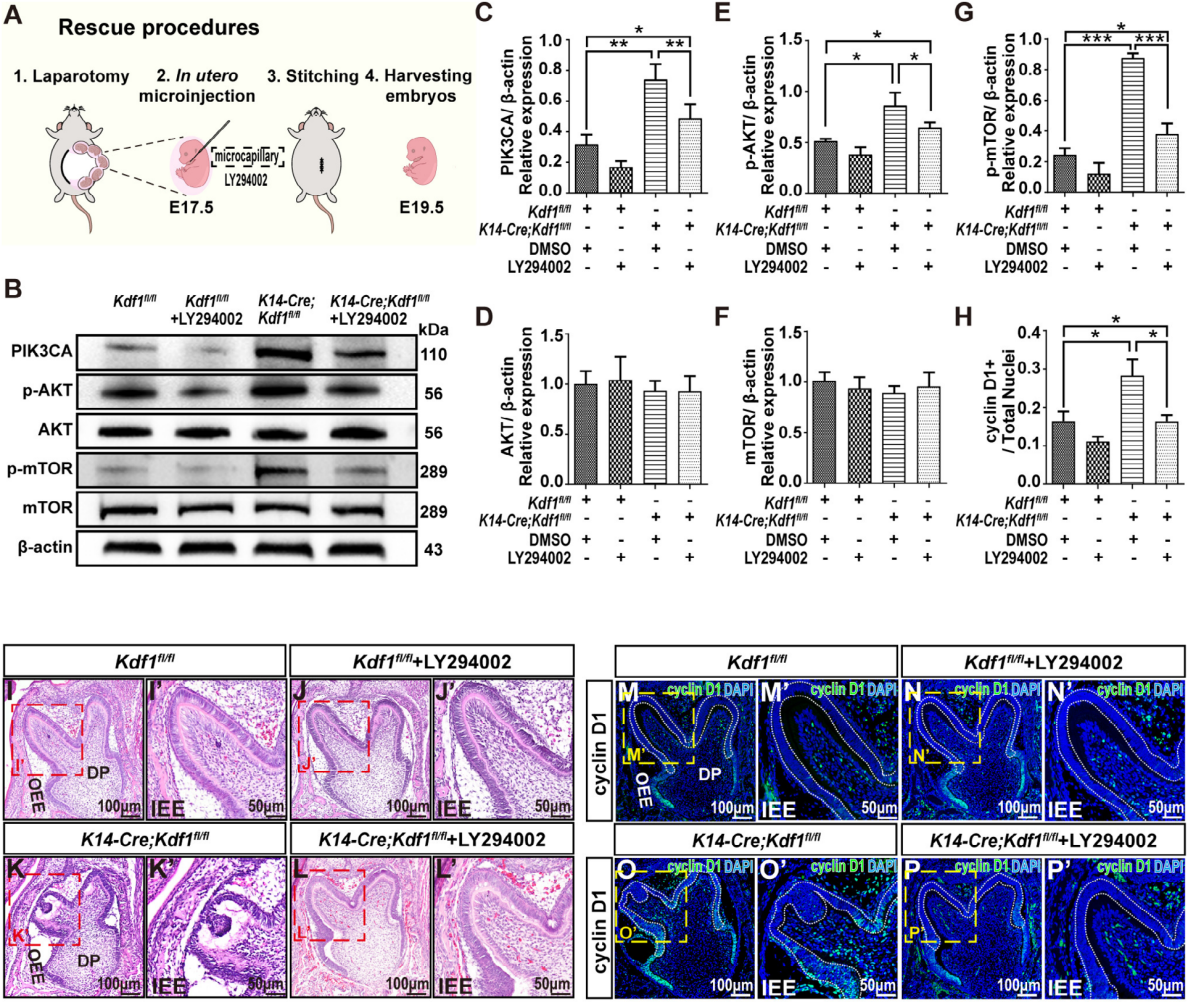
Pharmacological inhibition of PI3K/AKT/mTOR signalling with LY294002 decreases IEE proliferation and partially rescues cusp developmental defect in *K14‐Cre;Kdf1* ^*fl/fl*^ mice. (A) Diagrams of rescue procedures. (B‐G) Western blot results show the expression levels of PIK3CA, phosphor‐AKT (p‐AKT), AKT, phosphor‐mTOR (p‐mTOR), and mTOR in the IEE of the *Kdf1* ^*fl/fl*^ + DMSO, *Kdf1* ^*fl/fl*^ + LY294002, *K14‐Cre;Kdf1* ^*fl/fl*^ + DMSO, and *K14‐Cre;Kdf1* ^*fl/fl*^ + LY294002 groups. (I‐L) Representative coronal images of H&E‐stained MM1 from the *Kdf1* ^*fl/fl*^ + DMSO, *Kdf1* ^*fl/fl*^ + LY294002, *K14‐Cre;Kdf1* ^*fl/fl*^ + DMSO and *K14‐Cre;Kdf1* ^*fl/fl*^ + LY294002 groups at E19.5. Scale bars: 100 μm. (I′–L′) Higher magnifications of the representative cusp region boxed in red in I–L. Scale bars: 50 μm. (M–P) Co‐localizations of anti‐cyclin D1 (green) and DAPI (blue) exhibit the cyclin D1‐positive cells in MM1 from the *Kdf1* ^*fl/fl*^ + DMSO, *Kdf1* ^*fl/fl*^ + LY294002, *K14‐Cre;Kdf1* ^*fl/fl*^ + DMSO and *K14‐Cre;Kdf1* ^*fl/fl*^ + LY294002 groups at E19.5. The white dashed lines circle the cusp IEE. Scale bars: 100 μm. (M′–P′) Higher magnifications of the representative cusp region boxed in yellow in M‐P. The white dashed lines circle the cusp IEE. Scale bars: 50 μm. (H) Ratios of cyclin D1^+^/ DAPI^+^ represent the cell proliferation rate of the entire cusp IEE at E19.5. IEE, inner enamel epithelium; OEE, outer enamel epithelium; DP, dental pulp. Values are presented as mean ± SD. *p < 0.05, **p < 0.01, ***p < 0.001. *n* = 3 per group.

To verify whether the overactivation of PI3K/AKT/mTOR signalling was responsible for the increased IEE cell proliferation and cusp malformation in *K14‐Cre;Kdf1* ^*fl/fl*^ mice, we conducted in utero microinjection of LY294002, a PI3K/AKT/mTOR inhibitor, in the mandibular molar region of E17.5 embryos (Figure 6A). First, we injected an ink to indicate the application range of in utero microinjection of LY294002. The results showed that the mandibular first and second molar tooth germs were covered after injection (Figure S4A). Western blotting was performed to confirm the inhibitory effect of PI3K/AKT/mTOR signalling (Figure 6B). Compared to that of *K14‐Cre;Kdf1* ^*fl/fl*^ MM1 treated with DMSO, the expression level of PIK3CA and the phosphorylation levels of AKT and mTOR were significantly reduced in *K14‐Cre;Kdf1* ^*fl/fl*^ MM1 treated with LY294002 (Figure 6B–G), indicating that the overactivation of PI3K/AKT/mTOR signalling was partially alleviated. Furthermore, the disorganised, arranged IEE cells were partially restored and more pointed cusps were formed in LY294002‐treated MM1 in *K14‐Cre;Kdf1* ^*fl/fl*^ mice (Figure 6I–L,I′–L′), demonstrating that inhibition of the overactivated PI3K/AKT/mTOR pathway partially rescued the molar cusp defects in *K14‐Cre;Kdf1* ^*fl/fl*^ mice. Consistently, the expression level of cyclin D1 in IEE cells was significantly decreased in *K14‐Cre;Kdf1* ^*fl/fl*^ MM1 treated with LY294002, compared with that of *K14‐Cre;Kdf1* ^*fl/fl*^ MM1 treated with LY294002 (Figure 6H,M–P,M′–P′), indicating that the increased proliferation of IEE cells in the MM1 cusp of *K14‐Cre;Kdf1* ^*fl/fl*^ mice was partially recovered. Collectively, our results demonstrate that the suppression of PI3K/AKT/mTOR signalling partially inhibits the excessive proliferation of IEE cells in the molar cusps and rescues the cusp defect in *K14‐Cre;Kdf1* ^*fl/fl*^ mice.

## 4 Discussion

Understanding the function of genes associated with human tooth agenesis is crucial for the early detection and prevention of tooth developmental defects [40, 41]. For the first time, we generated *K14‐Cre;Kdf1* ^*fl/fl*^ mice, the molar malformation of which phenocopied the molar crown deformities of patients with *KDF1* variation, presenting with a rounded and blunt cusp morphology [18]. Our results revealed that the ablation of epithelial *Kdf1* resulted in the overactivation of the PI3K/AKT/mTOR signalling axis in IEE cells, leading to increased proliferation of IEE cells, subsequently resulting in the malformation of molar cusps.

The enamel knot and IEE play pivotal roles in the genesis of tooth cusps, and the functions of enamel knots in cusp development are relatively well documented [42, 43]. However, the intricacies of the IEE cellular behaviours and regulatory mechanisms underlying cusp patterning remain largely unknown. Previous studies revealed that a reduction in the proliferative activity of IEE cells during the late bell stage disrupts cusp morphology, leading to shallow folding and formation of non‐polarised, blunted cusps [15, 44]. Furthermore, it was observed that a decreased rate of IEE cell proliferation resulted in a lower cell layer count and diminished nuclear polarisation [15]. Additionally, we observed a marked increase in the activity of IEE cells following epithelial *Kdf1* knockout at the late bell stage. This hyperproliferation triggered the clustering of IEE cells into localised aggregates at the apex of molar cusps, consequently giving rise to the development of cusps that were both shallow and blunted. Both our and previous studies confirm that the precise regulation of IEE cell proliferation is critical for proper cusp morphogenesis.

*Kdf1* is a key regulatory factor in the development of ectoderm‐derived organs [16, 17, 18, 19, 21, 45]. In skin development, *Kdf1* is crucial for the proliferation–differentiation balance of epidermal progenitor cells [16]. In tooth development, *Kdf1* determines ameloblast differentiation, enamel volume, and mineral density [20, 21]. *Kdf1* promotes ameloblast differentiation by repressing the IKK/IκB/NF‐κB signalling axis in vitro [21]. In this study, we found increased proliferation and decreased differentiation of molar IEE cells in *K14‐Cre;Kdf1* ^*fl/fl*^ mice, indicating that epithelial *Kdf1* plays a crucial role in regulating IEE cell behaviour during cusp patterning.

The functional significance of PI3K/AKT signalling in the development of the dental mesenchyme has been extensively studied [46, 47, 48]. Recently, the importance of PI3K/AKT signalling in the development of dental epithelium has received considerable attention. PI3K/AKT signalling regulates epithelial stem cell activity and the proliferation of transit‐amplifying cells [49]. In miniature pig molar tooth germs, PI3K/AKT signalling is mainly expressed in the IEE of molar cusps at the early bell stage and in the odontoblasts at the late bell stage [26]. In this study, we found that the PI3K/AKT pathway was upregulated in IEE cells of molar cusps in *K14‐Cre;Kdf1* ^*fl/fl*^ mice. Therefore, *Kdf1* may govern the proliferation of IEE cells to modulate cusp morphology via the PI3K/AKT pathway.

mTOR, a crucial downstream target of AKT, is essential for cell growth and proliferation during embryogenesis [50, 51]. Epithelial mTOR is essential for molar cusp morphogenesis. mTOR‐deficient mice showed hypoplastic and atypical cusps and reduced cell proliferation during the bell stage [27]. In *K14‐Cre;Kdf1* ^*fl/fl*^ mice, p‐mTOR expression was upregulated in molar IEE cells, suggesting that the PI3K/AKT/mTOR signalling axis is involved in cusp morphogenesis. Furthermore, pharmacological inhibition of the overactivated PI3K/AKT/mTOR signalling pathway partially restored both the increased proliferation of IEE cells and morphological defects of molar cusps, demonstrating that the PI3K/AKT/mTOR cascade is a downstream effector of *Kdf1* in orchestrating the proliferation of IEE cells during cusp morphogenesis. While our study establishes that epithelial *Kdf1* ablation leads to overactivation of the PI3K/AKT/mTOR axis, the precise molecular mechanism requires further investigation. Given that KDF1 is a cytoplasmic protein lacking transmembrane domains or catalytic motifs [17, 21], it likely functions as a regulatory adaptor or scaffold protein rather than a direct signalling component. Based on the western blot data showing increased PIK3CA protein levels in *K14‐Cre;Kdf1* ^*fl/fl*^ IEE cells, we speculate that KDF1 may physically associate with PIK3CA (the catalytic subunit of PI3K) or its upstream regulators to modulate stability or activity. Previous studies have shown that KDF1 stabilises IKKα via deubiquitination during epidermal development [45], raising the possibility that it similarly regulates PIK3CA turnover. However, this proposed mechanism requires further experimental validation to determine whether KDF1 indeed modulates PI3K components via this interaction.

## 5 Conclusion

Taken together, our results provide the first in vivo evidence that *Kdf1* regulates the expression of the PI3K/AKT/mTOR signalling axis to control IEE cell proliferation during molar cusp patterning. Information from this study enhances our understanding of the molecular and cellular mechanisms underlying tooth development and may provide novel regulatory targets for tooth regeneration strategies.

## Author Contributions

**Jiayu Wang:** formal analysis, investigation, writing – original draft, visualisation. **Miao Yu:** conceptualisation, investigation, writing – original draft, funding acquisition. **Hangbo Liu:** validation, data curation. **Kai Sun:** validation, data curation. **Chenxin Geng:** validation, visualisation. **Haochen Liu:** validation, visualisation. **Hailan Feng:** project administration, funding acquisition. **Yang Liu:** formal analysis, supervision. **Hu Zhao:** formal analysis, supervision. **Dong Han:** conceptualisation, writing – review and editing, supervision, project administration, funding acquisition. All authors have approved the final version for submission.

## Ethics Statement

All animal study protocols were prepared before the study and approved by the Ethics Committee of the Peking University Health Science Center (LA2022177).

## Conflicts of Interest

The authors declare no conflicts of interest.

## Supporting information


**Data S1.** Supporting information.

## Data Availability

All RNA sequencing data are available from the Gene Expression Omnibus under the accession number: GSE268481 (https://www.ncbi.nlm.nih.gov/geo/query/acc.cgi?acc=GSE268481). Additional data are available upon request.
