# Supplementary material for: Kdf1 Regulates Molar Cusp Morphogenesis via the PI3K/AKT/mTOR Signalling Axis
Source: Cell Prolif. 2025 Jul 30;59(3):e70108. doi: 10.1111/cpr.70108 (PMC12961517; doi:10.1111/cpr.70108)
Supplement: Supplementary file 1 — Data S1. Supporting information. [file CPR-59-e70108-s001.docx]

**Supplementary materials**

***Kdf1* regulates molar cusp morphogenesis via the PI3K/AKT/mTOR signaling axis**

Jiayu Wang^1†^, Miao Yu^1†^, Hangbo Liu^1^, Kai Sun^1^, Chenxin Geng^1^, Haochen Liu^1^, Hailan Feng^1^, Yang Liu^1^, Hu Zhao^2^, Dong Han^1*^

**Appendix Materials and Methods**

***transgenic mice genotyping***

The mouse offspring were genotyped using a One Step Mouse Genotyping Kit (Vazyme, Nanjing, China). Specifically, mouse tails or nails were digested by proteinase K for 20 min at 55°C. Subsequently, the temperature was adjusted to 95°C for 10 min to inactivate proteinase K. The four primer pairs were used for K14-Cre transgenic mice genotyping (control: 324 bp; mutant: 481 bp): Control-F: 5′-CTAGGCCACAGAATTGAAAGATCT-3′, Control-R: 5′-GTAGGTGGAAATTCTAGCATCATCC-3′, Cre-F: 5′-GCCTGCATTACCGGTCGATGC-3′, Cre-R: 5′-CAGGGTGTTATAAGCAATCCC-3′. Furthermore, two primer pairs were used for Kdf1-flox transgenic mice genotyping (wild type: 142 bp; mutant: 216 bp): Flox-F: 5'-AGTTTCCTAATGGACACTACAGGAC-3', Flox-R: 5'-CATGCTAGGCAGCACTATGGAC-3'.

***Micro-computed tomography (Micro-CT)***

Histomorphometry was conducted on the mandibular first molar after renal subcapsular development using Siemens Inveon micro-CT (Siemens Medical Solutions USA, Inc., Malvern, PA, USA) at 60 kV and 33 pixels. The 3D images were reconstructed using Avatar software (PINGSENG Healthcare, Kunshan, China).

***EdU (5-ethynyl-2'-deoxyuridine) incorporation and staining***

EdU (25 mg/g; RiboBio, Guangzhou, China) was intraperitoneally injected into pregnant mice (13.5, 15.5, 16.5, 17.5 and 18.5 days after the vaginal seminal plug was observed) or newborn mice 3 h prior to euthanization. After euthanasia, the heads of the embryos and newborn mice were fixed in a 4% PFA solution overnight at 4°C, followed by decalcification, dehydration, and sectioning. EdU signals were detected using a staining kit (RiboBio, C10371) according to the manufacturer's instructions. Keratin 14 immunofluorescence staining was performed to distinguish the dental epithelium from other parts. The EdU+/ DAPI+ nuclei ratio was used to analyze cell proliferation ability.

***Immunofluorescence assays***

Paraffin sections at 5 µm from embryos (E13.5, E15.5, E16.5, E17.5 and E18.5) and newborn mice were deparaffinized with xylene and rehydrated through graded ethanol. After heat-induced antigen retrieval using citrate buffer, the sections were blocked for 1 h at room temperature in a blocking solution (ZSGB-BIO, Beijing, China). Subsequently, sections were separately incubated with primary antibodies against Keratin 14 (1:100; abcam, Cambridge, UK), cyclin D1 (1:100; Cell Signaling Technology, Danvers, USA), and amelogenin (1:100; abcam, Cambridge, UK) overnight at 4°C. After washing with PBS, the slides were incubated with fluorescently labeled secondary antibodies (Zhongshan Golden Bridge Biotechnology, Beijing, China; 1:200) for 2 h at room temperature. DAPI (ZSGB-BIO) was used for nuclear staining. All images were acquired using a confocal microscope (Leica SP8; Wetzlar, Germany).

***RNA-seq and data analysis***

RNA samples from the IEE of the mandibular first molars were collected from *Kdf1^fl/fl^* and *K14-Cre;Kdf1 ^fl/fl^* mice at the E18.5 stage (*n* = 3 per group). mRNA extraction, cDNA library construction, and sequencing were performed at BGI Genomics Co., Ltd. (Shenzhen, China). The absolute value of log2 fold change ≥ 1 and *Q* value ≤ 0.05 were used as criteria to identify the differential expression genes. KEGG enrichment of annotated differentially expressed genes was supported by the online bioinformatics platform of Dr. Tom (https://biosys.bgi.com/) from the Beijing Genomics Institute.

***Western blot***

Total protein was extracted from the IEE using RIPA lysis buffer (Beyotime, Shanghai, China). Proteins were separated by sodium dodecyl sulfate-polyacrylamide gel electrophoresis (SDS-PAGE; ACE Biotechnology, Nanjing, China) and transferred onto PVDF membranes (Millipore, Massachusetts, USA). Defatted milk (5%) was used for 2 h at room temperature for blocking. The membranes were incubated with primary antibody against Kdf1 (1:500; abcam, Cambridge, UK), AKT (1:1000; abcam, Cambridge, UK), p-AKT (Ser473) (1:5000; abcam, Cambridge, UK), mTOR (1:5000; abcam, Cambridge, UK), p-mTOR (Ser2448) (1:1000; abcam, Cambridge, UK), PIK3CA (1:1000; Cell Signaling Technology, Danvers, USA), GSK3β (1:1000; abcam, Cambridge, UK), p-GSK3β (Ser9) (1:5000; abcam, Cambridge, UK), FOXO3a (1:1000; Cell Signaling Technology, Danvers, USA), p-FOXO3a (S253) (1:1000; Abclonal, Wuhan, China), or β-actin (1 : 5000; Proteintech, Chicago, USA) overnight at 4°C. After washing and incubation with secondary antibody, bands were detected using enhanced chemiluminescence (Tanon, Shanghai, China). Densitometric quantification of the protein bands was performed using ImageJ software (Bethesda, Maryland, USA).

**Supplementary figures**


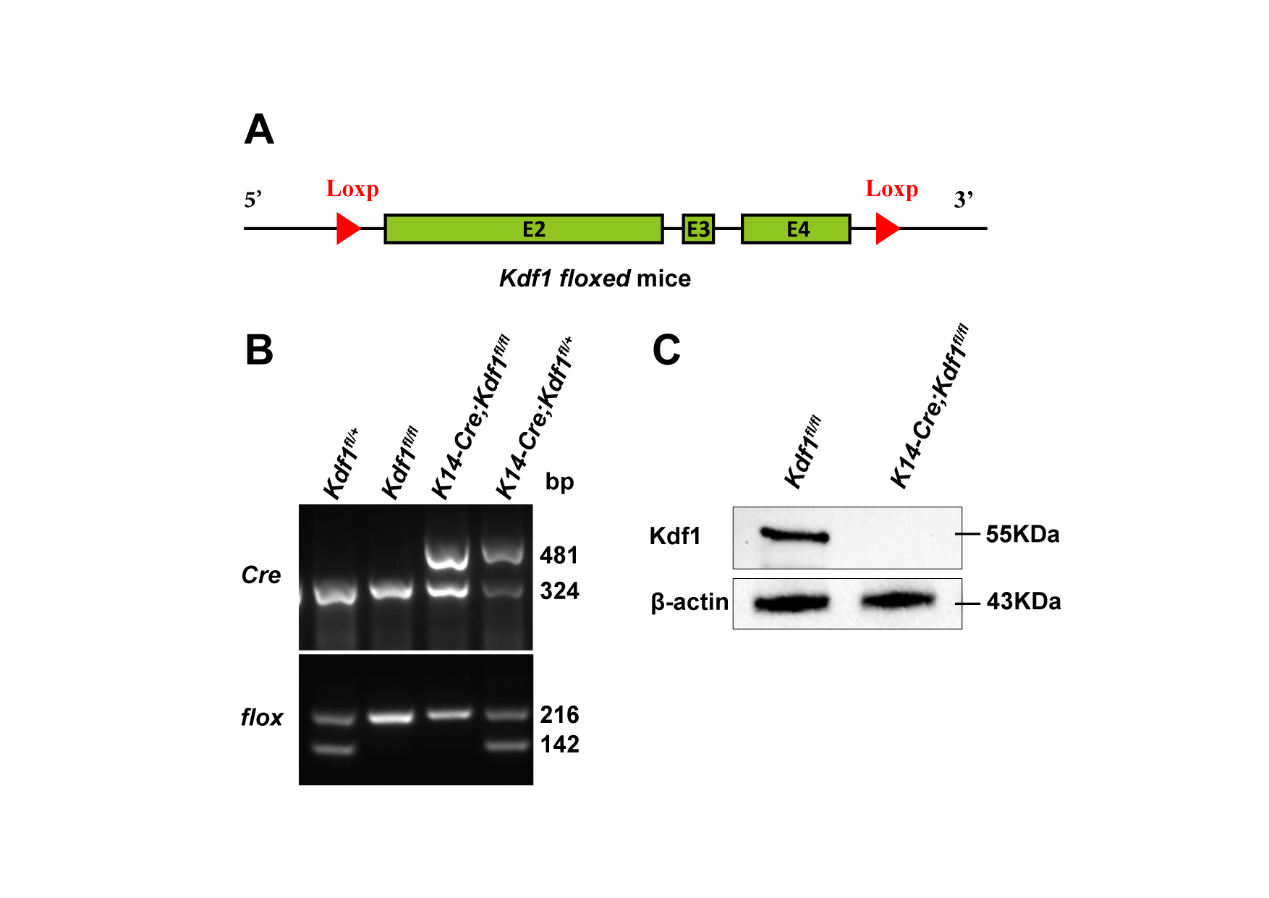


**Figure S1. Construction of epithelial *Kdf1* knockout mice using the *K14-Cre* transgene.** (**A**) The developing strategy of *Kdf1 floxed* mice. (**B**) PCR genotyping of *K14-Cre* transgenic and *floxed* alleles. (**C**) Western blot analysis of Kdf1 in the dental epithelium of control *Kdf1^fl/fl^* and *K14-Cre;Kdf1^fl/fl^* mice.


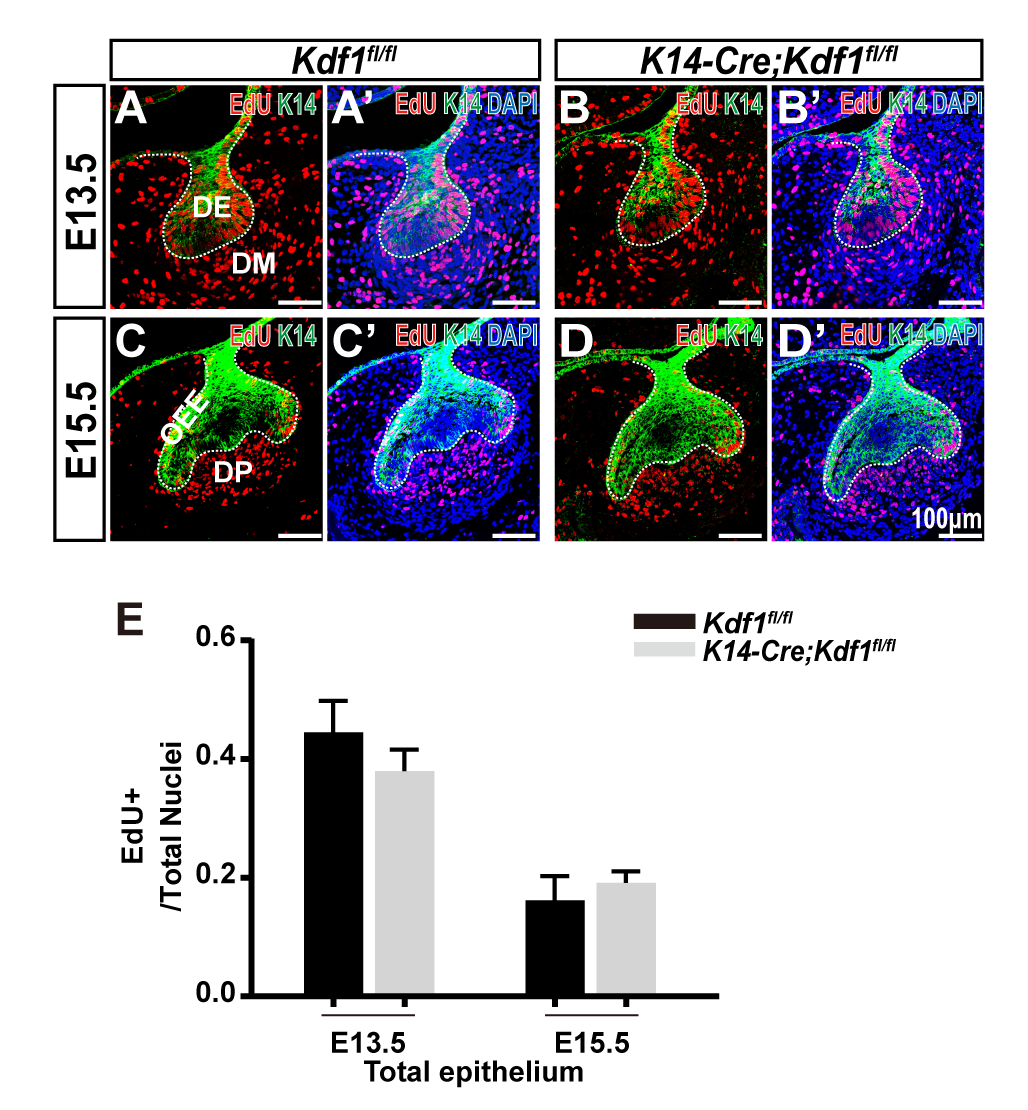


**Figure S2. Unaffected proliferation ability of dental epithelium in *K14-Cre;Kdf1^fl/fl^* mice at E13.5 and E15.5.** (**A-D and A’-D’**) Combinations of three fluorescence signals (anti-K14 in green, EdU in red, and DAPI in blue) show EdU-positive cells of MM1 in *Kdf1^fl/fl^* and *K14-Cre;Kdf1^fl/fl^* mice at E13.5 and E15.5. The white dashed lines indicate dental epithelium. Scale bars: 100 μm. (**E**) Ratios of EdU^+^/ DAPI^+^ cells exhibiting cell proliferation rate in the entire crown epithelium at E13.5 and E15.5. DE, dental epithelium; DM, dental mesenchyme; OEE, outer enamel epithelium; DP, dental pulp. Values are presented as mean ± SD. *n*=3 per group.


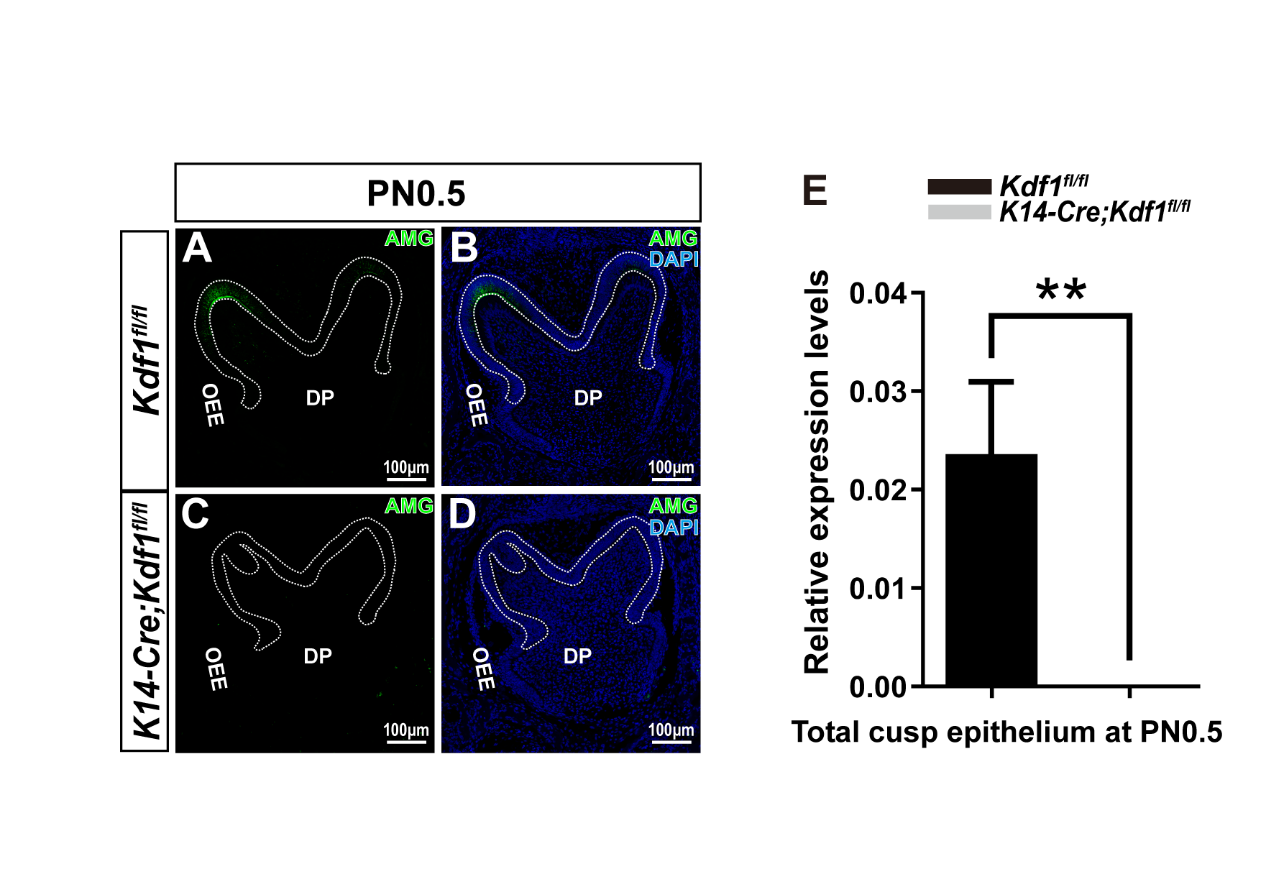


**Figure S3. Decreased differentiation capacity of IEE in *K14-Cre;Kdf1^fl/fl^* mice at PN0.5.** (**A-D**) Combinations of two fluorescence signals (anti-AMG in green and DAPI in blue) exhibiting AMG-positive cells in MM1 from *Kdf1^fl/fll^* and *K14-Cre;Kdf1^fl/fll^* mice at PN0.5. The white dashed lines circle the cusp IEE. Scale bars: 100 μm. (**E**) Fluorescence density analysis of AMG in the entire cusp IEE at PN0.5. Values are presented as mean ± SD. ***P*<0.01. *n*=3 per group.


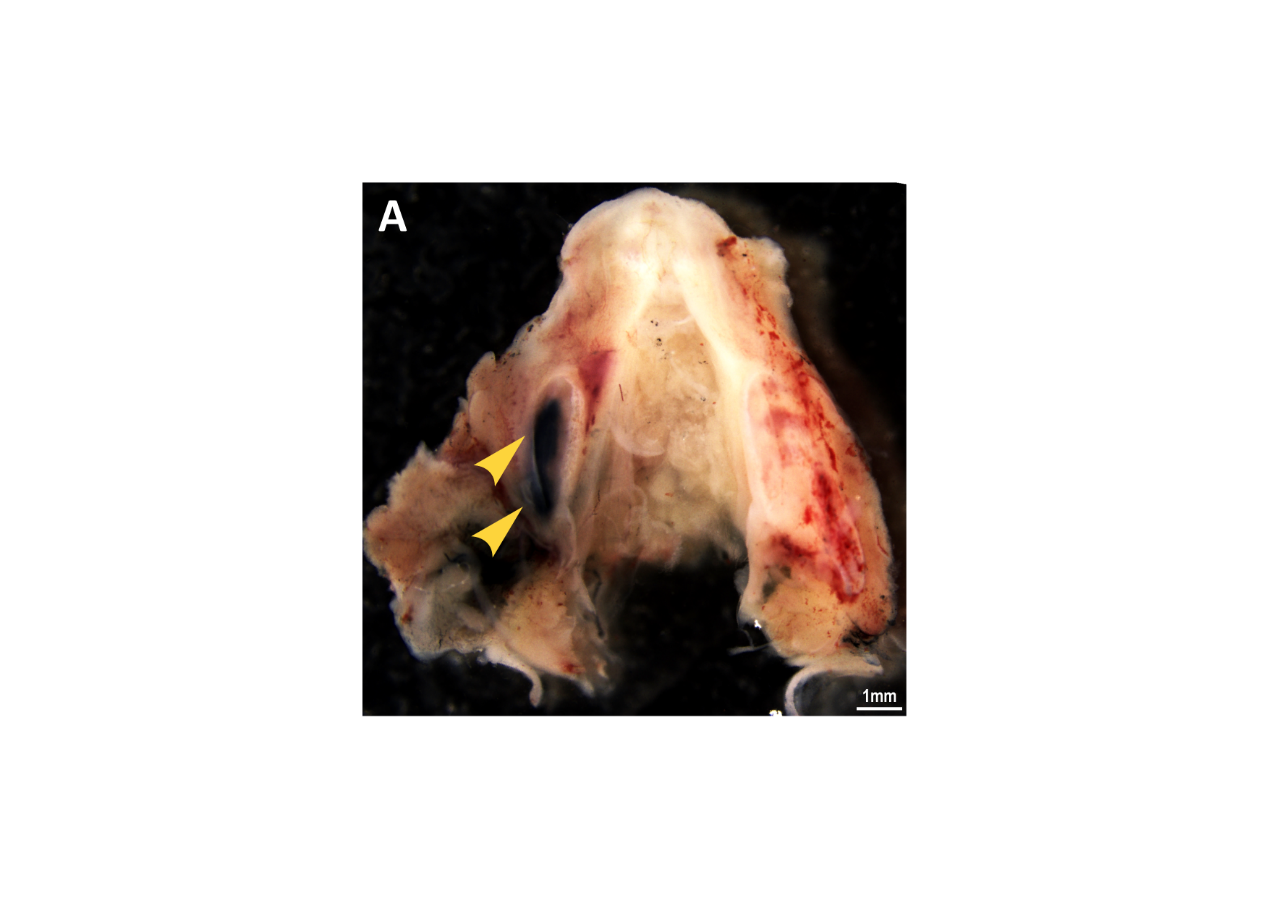


**Figure S4. The coverage of *in utero* microinjection with microcapillary.** (**A**) Stereomicroscopic view of the mandible of E17.5 mouse embryo after *in utero* microinjection of ink with microcapillaries. Arrowheads indicate labeled mandibular first molar (MM1) and mandibular second molar (MM2) with ink. Scale bar: 1 mm.
